# Supplementary material for: Effects of Roughage on the Lipid and Volatile-Organic-Compound Profiles of Donkey Milk
Source: Foods. 2023 Jun 1;12(11):2231. doi: 10.3390/foods12112231 (PMC10252442; doi:10.3390/foods12112231)
Supplement: Supplementary file 1 [file foods-12-02231-s001.zip › foods-2416170-supplementary.pdf]

Supplementary Figures

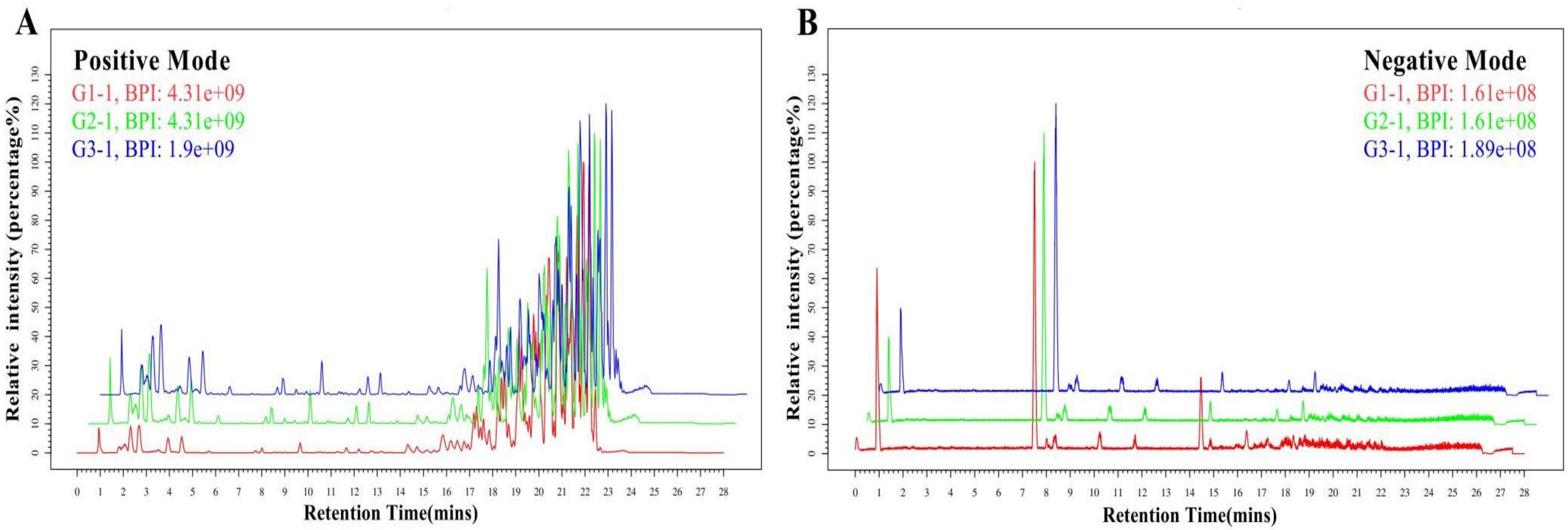

Figure S1. The positive (A) and negative mode (B) of lipid base peak chromatogram in donkey milk.

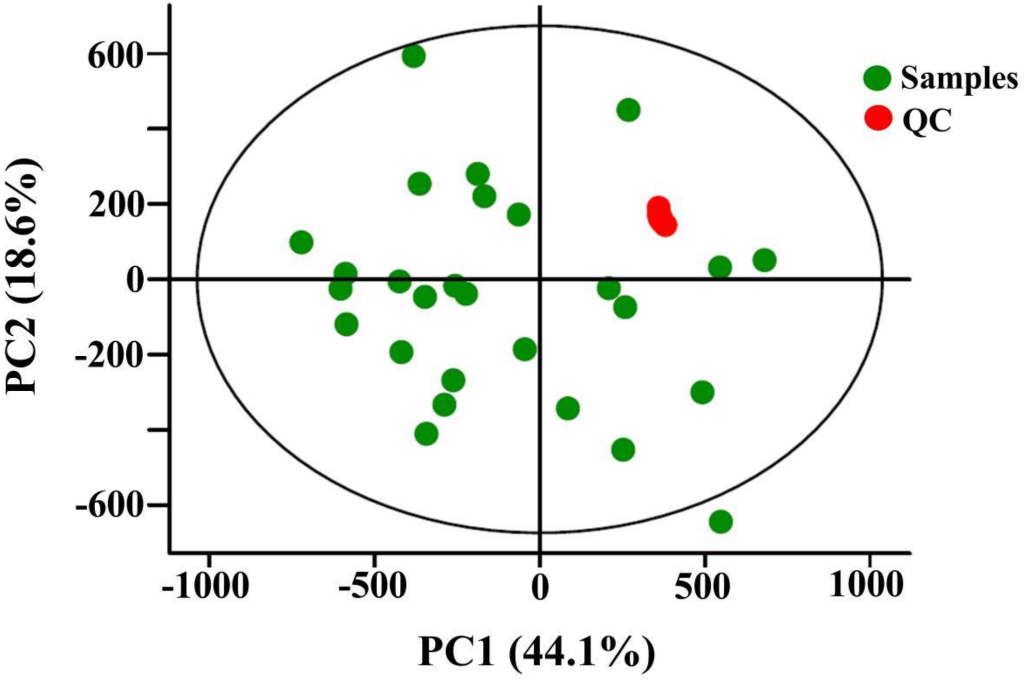

Figure S2. PCA score chart of donkey milk lipid quality control (QC).

Supplementary Tables

Table S1. Differential lipids in donkey milk fed on different roughages.

No.	Lipid	Class	Category	MainIon	mz	Retention time (min)	VIP	*p*-value
1	WE(2:0_19:4)	WE	FAs	M+NH4	336.2897	7.876	2.06	0.0208
2	Cer(d18:1_16:0)	Cer	SPs	M+H	538.5194	17.305	1.24	0.0000
3	Cer(d16:0_18:0)	Cer	SPs	M+H	540.535	15.158	1.01	0.0022
4	Cer(d36:0)	Cer	SPs	M+H	568.5663	16.779	1.52	0.0022
5	Cer(d38:0)	Cer	SPs	M+H	596.5976	18.09	1.47	0.0035
6	Cer(d37:0)	Cer	SPs	M+H	582.582	17.552	2.12	0.0036
7	Cer(d22:0_18:0)	Cer	SPs	M+H	624.6289	19.094	1.08	0.0036
8	SM(d40:0)	SM	SPs	M+H	789.6844	19.281	1.83	0.0013
9	SM(d40:1)	SM	SPs	M+H	787.6688	19.002	1.29	0.0017
10	SM(d41:0)	SM	SPs	M+H	803.7001	19.604	1.14	0.0044
11	SM(d42:0)	SM	SPs	M+H	817.7157	19.909	1.43	0.0158
12	BisMePA(20:2e_12:0)	BisMePA	GPs	M+Na	681.483	17.228	1.72	0.0014
13	BisMePA(30:1_16:0)	BisMePA	GPs	M+Na	893.697	21.637	1.15	0.0058
14	BisMePA(30:0_16:0)	BisMePA	GPs	M+Na	895.7126	21.977	1.10	0.0089
15	MePC(33:1)	MePC	GPs	M+Na	782.567	15.057	1.09	0.0001
16	MePC(35:1)	MePC	GPs	M+Na	810.5983	17.19	2.07	0.0001

17	MePC(30:0)	MePC	GPs	M+Na	742.5357	17.007	2.08	0.0002
18	PC(33:3)	PC	GPs	M+H	742.5381	16.528	1.52	0.0007
19	PC(44:0e)	PC	GPs	M+H	888.778	21.632	1.62	0.0045
20	PE(18:1_18:2)	PE	GPs	M+H	742.5381	16.514	1.90	0.0004
21	MG(18:2)	MG	GLs	M+H	355.2843	19.124	1.17	0.0171
22	DG(24:1e)	DG	GLs	M+Na	463.3758	17.379	1.00	0.0011
23	DG(24:0e)	DG	GLs	M+Na	465.3914	19.047	1.22	0.0018
24	DG(26:3e)	DG	GLs	M+H	465.3938	18.948	1.21	0.0037
25	DG(18:0_16:0)	DG	GLs	M+NH4	614.5718	19.547	1.89	0.0042
26	DG(18:0_18:0)	DG	GLs	M+NH4	642.6031	20.141	1.37	0.0048
27	DG(16:0_16:0)	DG	GLs	M+NH4	586.5405	18.839	1.10	0.0079
28	TG(16:0_8:0_20:4)	TG	GLs	M+NH4	760.645	20.215	8.89	0.0000
29	TG(8:0_14:2_18:1)	TG	GLs	M+NH4	706.598	19.702	3.20	0.0000
30	TG(8:0_14:0_20:4)	TG	GLs	M+NH4	732.6137	19.741	3.21	0.0000
31	TG(8:0_10:0_18:3)	TG	GLs	M+NH4	650.5354	18.015	4.76	0.0000
32	TG(6:0_10:0_18:2)	TG	GLs	M+NH4	624.5198	16.062	2.49	0.0000
33	TG(10:0_14:0_17:1)	TG	GLs	M+NH4	724.645	20.797	1.12	0.0000
34	TG(16:0_8:0_12:2)	TG	GLs	M+Na	657.5065	18.642	2.67	0.0000
35	TG(8:0_10:1_18:3)	TG	GLs	M+NH4	648.5198	16.897	1.12	0.0000
36	TG(8:0_10:0_14:1)	TG	GLs	M+Na	603.4595	17.322	1.02	0.0001
37	TG(18:0_8:0_8:0)	TG	GLs	M+NH4	628.5511	17.646	1.21	0.0001
38	TG(16:0_10:0_18:1)	TG	GLs	M+NH4	766.6919	21.142	3.11	0.0001

39	TG(4:0_16:0_18:3)	TG	GLs	M+NH4	678.5667	18.899	3.06	0.0001
40	TG(8:0_12:0_20:4)	TG	GLs	M+NH4	704.5824	19.169	1.40	0.0001
41	TG(16:0_8:0_16:1)	TG	GLs	M+NH4	710.6293	20.521	11.33	0.0001
42	TG(10:0_10:1_20:5)	TG	GLs	M+H	683.5245	18.649	1.14	0.0001
43	TG(16:0_12:0_18:1)	TG	GLs	M+NH4	794.7232	21.512	2.90	0.0001
44	TG(10:0_12:0_18:1)	TG	GLs	M+NH4	710.6293	20.226	2.85	0.0001
45	TG(8:0_10:1_18:2)	TG	GLs	M+NH4	650.5354	17.694	3.82	0.0002
46	TG(4:0_12:0_18:3)	TG	GLs	M+H	605.4776	18.353	1.97	0.0002
47	TG(16:0_10:0_20:4)	TG	GLs	M+NH4	788.6763	20.705	8.19	0.0002
48	TG(6:0_10:2_23:0)	TG	GLs	M+NH4	694.598	19.037	1.22	0.0002
49	TG(10:0_12:0_18:3)	TG	GLs	M+NH4	706.598	19.3	5.28	0.0002
50	TG(8:0_10:3_14:1)	TG	GLs	M+H	575.4306	16.133	1.11	0.0002
51	TG(16:0_10:0_16:0)	TG	GLs	M+NH4	740.6763	21.144	5.61	0.0002
52	TG(10:0_10:0_18:3)	TG	GLs	M+NH4	678.5667	17.673	2.61	0.0002
53	TG(10:0_10:0_12:2)	TG	GLs	M+NH4	596.4885	14.989	1.51	0.0002
54	TG(8:0_10:2_18:2)	TG	GLs	M+H	631.4932	15.283	1.05	0.0002
55	TG(45:7)	TG	GLs	M+NH4	768.6137	20.168	2.15	0.0003
56	TG(12:0_18:2_18:2)	TG	GLs	M+NH4	816.7076	21.136	6.2	0.0003
57	TG(18:1_10:1_17:1)	TG	GLs	M+NH4	776.6763	20.873	2.29	0.0004
58	TG(16:0_6:0_8:0)	TG	GLs	M+NH4	572.4885	17.353	8.74	0.0004
59	TG(16:0_17:1_18:2)	TG	GLs	M+H	843.7436	20.411	1.48	0.0004
60	TG(18:1_12:0_18:1)	TG	GLs	M+NH4	820.7389	21.512	3.59	0.0004

61	TG(16:1_8:0_10:0)	TG	GLs	M+NH4	626.5354	18.287	6.35	0.0005
62	TG(8:0_8:0_20:5)	TG	GLs	M+H	629.4776	17.285	1.07	0.0007
63	TG(6:0_18:2_18:2)	TG	GLs	M+NH4	732.6137	19.37	3.40	0.0007
64	TG(18:4_8:0_18:1)	TG	GLs	M+NH4	758.6293	19.815	1.77	0.0007
65	TG(50:1)	TG	GLs	M+NH4	850.7858	21.819	3.84	0.0008
66	TG(18:1_18:3_20:5)	TG	GLs	M+H	901.728	21.269	1.04	0.0008
67	TG(4:0_10:0_12:0)	TG	GLs	M+Na	521.3813	14.499	1.01	0.0008
68	TG(8:0_11:4_18:2)	TG	GLs	M+Na	663.4595	17.11	9.28	0.0009
69	TG(39:9)	TG	GLs	M+H	663.4619	17.241	9.14	0.0009
70	TG(10:0_10:0_18:1)	TG	GLs	M+NH4	682.598	19.69	4.34	0.0009
71	TG(16:0_12:0_16:0)	TG	GLs	M+NH4	768.7076	21.517	4.05	0.0009
72	TG(8:0_8:0_18:2)	TG	GLs	M+Na	629.4752	17.449	1.27	0.0011
73	TG(10:0_10:0_10:1)	TG	GLs	M+NH4	570.4728	14.955	1.84	0.0012
74	TG(18:4_8:0_16:0)	TG	GLs	M+NH4	732.6137	18.804	1.58	0.0014
75	TG(16:0_18:3_18:3)	TG	GLs	M+NH4	868.7389	21.238	3.04	0.0014
76	TG(8:0_10:0_18:1)	TG	GLs	M+NH4	654.5667	19.036	5.94	0.0014
77	TG(18:2_10:1_18:2)	TG	GLs	M+NH4	786.6606	20.303	2.89	0.0017
78	TG(18:1_14:0_20:4)	TG	GLs	M+H	853.728	21.782	1.71	0.0017
79	TG(8:0_10:0_18:2)	TG	GLs	M+NH4	652.5511	18.385	6.06	0.0018
80	TG(8:0_18:2_18:2)	TG	GLs	M+NH4	760.645	19.912	4.63	0.0018
81	TG(12:0_18:2_18:3)	TG	GLs	M+NH4	814.6919	20.494	2.51	0.0018
82	TG(16:0_18:2_20:5)	TG	GLs	M+H	877.728	21.606	1.97	0.0021

83	TG(18:1_12:0_18:3)	TG	GLs	M+NH4	816.7076	20.854	1.57	0.0021
84	TG(8:0_17:1_18:2)	TG	GLs	M+NH4	748.645	20.111	1.76	0.0023
85	TG(16:1_8:0_18:2)	TG	GLs	M+NH4	734.6293	19.882	5.86	0.0023
86	TG(15:0_8:0_18:2)	TG	GLs	M+NH4	722.6293	20.082	1.91	0.0023
87	TG(15:0_8:0_18:3)	TG	GLs	M+NH4	720.6137	19.578	1.02	0.0025
88	TG(16:0_18:2_20:4)	TG	GLs	M+H	879.7436	21.888	2.09	0.0025
89	TG(4:0_16:0_18:2)	TG	GLs	M+Na	685.5378	19.432	2.49	0.0025
90	TG(53:7)	TG	GLs	M+NH4	880.7389	21.879	1.60	0.0027
91	TG(18:1_18:3_18:3)	TG	GLs	M+NH4	894.7545	20.995	1.48	0.0027
92	TG(20:0_18:3_18:3)	TG	GLs	M+NH4	924.8015	21.313	2.48	0.0031
93	TG(10:0_18:1_18:1)	TG	GLs	M+NH4	792.7076	21.174	3.19	0.0031
94	TG(8:0_8:0_18:1)	TG	GLs	M+NH4	626.5354	17.296	1.22	0.0033
95	TG(16:0_18:1_21:1)	TG	GLs	M+NH4	918.8484	22.239	1.15	0.0035
96	TG(10:0_18:2_18:2)	TG	GLs	M+NH4	788.6763	20.393	3.44	0.0036
97	TG(41:6e)	TG	GLs	M+Na	705.5428	20.213	1.19	0.0037
98	TG(12:0_10:1_18:3)	TG	GLs	M+NH4	704.5824	18.774	2.14	0.0038
99	TG(18:0e_6:0_12:0)	TG	GLs	M+NH4	642.6031	19.53	1.72	0.0042
100	TG(16:0_8:0_16:0)	TG	GLs	M+NH4	712.645	20.712	4.60	0.0043
101	TG(18:0_10:0_18:0)	TG	GLs	M+NH4	796.7389	21.494	2.80	0.0045
102	TG(55:4)	TG	GLs	M+NH4	914.8171	21.699	1.40	0.0051
103	TG(55:8e)	TG	GLs	M+Na	897.7306	22.088	1.06	0.0051
104	TG(18:3_17:1_20:5)	TG	GLs	M+NH4	904.7389	21.618	1.03	0.0053

105	TG(16:0_14:0_18:3)	TG	GLs	M+Na	823.6786	21.247	1.24	0.0055
106	TG(10:0_12:0_22:0)	TG	GLs	M+NH4	768.7076	21.124	3.83	0.0058
107	TG(18:1_18:2_20:2)	TG	GLs	M+NH4	926.8171	21.948	1.10	0.0064
108	TG(8:0_12:0_12:0)	TG	GLs	M+NH4	600.5198	16.986	4.58	0.0066
109	TG(18:1_12:0_20:4)	TG	GLs	M+H	825.6967	21.541	1.28	0.0070
110	TG(20:3_18:2_18:2)	TG	GLs	M+NH4	922.7858	21.038	1.49	0.0074
111	TG(16:1_10:0_20:4)	TG	GLs	M+H	769.6341	20.748	2.11	0.0077
112	TG(10:0_18:1_20:4)	TG	GLs	M+H	797.6654	21.175	1.78	0.0078
113	TG(15:0_8:0_18:1)	TG	GLs	M+NH4	724.645	20.511	2.26	0.0079
114	TG(8:0_18:2_18:3)	TG	GLs	M+NH4	758.6293	19.477	3.01	0.0080
115	TG(8:0_17:1_18:1)	TG	GLs	M+NH4	750.6606	20.557	2.56	0.0089
116	TG(10:0_10:0_14:0)	TG	GLs	M+NH4	628.5511	18.057	5.78	0.0091
117	TG(37:6e)	TG	GLs	M+Na	649.4802	19.002	1.21	0.0107
118	TG(18:0_10:0_12:0)	TG	GLs	M+NH4	712.645	20.214	4.49	0.0108
119	TG(18:0_8:0_12:0)	TG	GLs	M+NH4	684.6137	19.654	5.10	0.0112
120	TG(14:0_14:0_14:0)	TG	GLs	M+NH4	740.6763	20.717	4.08	0.0112
121	TG(16:1_11:4_16:1)	TG	GLs	M+NH4	742.598	20.29	1.40	0.0119
122	TG(8:0_13:0_18:2)	TG	GLs	M+NH4	694.598	19.509	1.23	0.0126
123	TG(15:0_18:1_18:3)	TG	GLs	M+NH4	858.7545	21.423	1.09	0.0131
124	TG(10:0_12:0_17:1)	TG	GLs	M+NH4	696.6137	19.995	1.64	0.0132
125	TG(45:8e)	TG	GLs	M+Na	757.5741	20.3	1.29	0.0133
126	TG(18:2_18:2_20:4)	TG	GLs	M+H	903.7436	21.558	1.26	0.0136

127	TG(12:0_12:0_20:4)	TG	GLs	M+H	743.6184	20.69	1.61	0.0141
128	TG(18:1_14:1_20:4)	TG	GLs	M+H	851.7123	21.59	1.33	0.0144
129	TG(16:1_13:0_18:2)	TG	GLs	M+NH4	804.7076	20.999	1.19	0.0158
130	TG(47:7e)	TG	GLs	M+Na	787.6211	21.109	1.04	0.0168
131	TG(10:0_10:1_17:1)	TG	GLs	M+NH4	666.5667	18.825	1.02	0.0171
132	TG(12:0_12:0_20:5)	TG	GLs	M+H	741.6028	20.292	1.96	0.0171
133	TG(47:8e)	TG	GLs	M+Na	785.6054	20.775	1.31	0.0172
134	TG(8:0_8:0_20:2)	TG	GLs	M+NH4	652.5511	17.389	2.24	0.0173
135	TG(18:1_18:1_18:3)	TG	GLs	M+NH4	898.7858	21.273	2.09	0.0188
136	TG(16:0_18:1_19:0)	TG	GLs	M+NH4	892.8328	22.252	1.10	0.0194
137	TG(10:0_12:0_20:5)	TG	GLs	M+H	713.5715	19.724	1.01	0.0210
138	TG(16:1_18:1_18:1)	TG	GLs	M+NH4	874.7858	21.873	7.09	0.0233
139	TG(16:0_16:1_18:1)	TG	GLs	M+NH4	848.7702	21.834	4.89	0.0242
140	TG(16:0e_6:0_16:0)	TG	GLs	M+NH4	670.6344	20.119	1.29	0.0251
141	TG(14:0_18:2_18:3)	TG	GLs	M+NH4	842.7232	20.923	2.04	0.0290
142	TG(16:1_10:0_18:1)	TG	GLs	M+NH4	764.6763	20.783	3.13	0.0336
143	TG(10:0_12:0_23:0)	TG	GLs	M+NH4	782.7232	21.294	1.17	0.0344
144	TG(10:0_12:0_18:2)	TG	GLs	M+NH4	708.6137	19.767	4.37	0.0356
145	TG(47:1)	TG	GLs	M+NH4	808.7389	21.355	1.02	0.0368
146	TG(26:1_8:0_10:0)	TG	GLs	M+NH4	766.6919	20.783	4.03	0.0382
147	TG(8:0_18:1_18:1)	TG	GLs	M+NH4	764.6763	20.323	3.6	0.0390
148	TG(10:0_18:1_20:2)	TG	GLs	M+NH4	818.7232	20.829	2.61	0.0409

149	TG(16:0_6:0_16:0)	TG	GLs	M+NH4	684.6137	20.199	3.21	0.0412
150	TG(10:0_18:1_20:3)	TG	GLs	M+NH4	816.7076	20.462	2.51	0.0412
151	TG(18:0_8:0_15:0)	TG	GLs	M+NH4	726.6606	20.522	1.17	0.0413
152	TG(17:0_10:0_18:2)	TG	GLs	M+NH4	778.6919	20.952	1.82	0.0443
153	TG(47:2)	TG	GLs	M+NH4	806.7232	21.001	1.30	0.0492

Abbreviations: mz, mass-to-chargeratio; VIP, variable importance in projection; TG, triglyceride; DG, diglyceride; MG, monoglyceride; MePC, methyl phosphatidylcholine; PC, phosphatidylcholine; PE, phosphatidylethanolamine; Cer, ceramide; SM, sphingomyelin; WE, wax ester; BisMePA, bis-methyl phosphatidic acid; FAs, fat acids; GLs, glycerolipids; GPs, glycerophospholipids; SPs, sphingolipids.

Table S2. Formation of volatile organic compounds in donkey milk for different roughage.

No.	Compounds (×10^5)	RI	NIST_RI	CAS	Match Factor	G1	G2	G3	*p* value
1	1-methyl-1-hydroxymethyladamantane	1383.95	1270	1200-78-8	67.26	0.19±0.07^b	0.78±0.11^a	1.00±0.19^a	0.001
2	1,3-dimethyl-benzene	868.48	907	108-38-3	75.78	0.47±0.03	0.47±0.02	0.49±0.02	0.810
3	1-ethyl-2,4-dimethyl-benzene	1152.85	1119	874-41-9	68.32	0.11±0.02^b	0.25±0.01^a	0.22±0.02^a	0.000
4	2,3,6-trimethyl-decane	1305.47	1121	62238-12-4	83.28	0.24±0.03	0.32±0.02	0.24±0.03	0.065
5	3-ethyl-3-methyl-decane	1260.55	1229	17312-66-2	90.8	0.91±0.09	1.03±0.10	0.85±0.09	0.421
6	6-ethyl-2-methyl-decane	1410.1	1185	62108-21-8	80.3	0.39±0.05^b	0.59±0.03^a	0.46±0.05^{ab}	0.012
7	dodecane	1198.9	1214	112-40-3	90.55	1.50±0.20	1.29±0.13	1.12±0.10	0.221
8	4,6-dimethyl-dodecane	1273.45	1285	61141-72-8	93.53	2.10±0.21	2.46±0.18	2.13±0.21	0.397
9	2-methyl-nonane	965.54	951	871-83-0	76.1	0.21±0.01^a	0.17±0.01^b	0.19±0.01^{ab}	0.036
10	3,3-dimethyl-octane	1021.18	931	4110-44-5	86.48	0.78±0.04	0.66±0.05	0.72±0.03	0.099
11	tetradecane	1399.15	1413	629-59-4	96.78	0.86±0.12^b	1.29±0.08^a	1.08±09^{ab}	0.017
12	2,8-dimethyl-undecane	1220.17	1185	17301-25-6	89.03	0.47±0.04	0.48±0.05	0.40±0.04	0.362
13	3,3-dimethyl-undecane	1419.97	1229	17312-65-1	77.21	0.11±0.02^b	0.18±0.01^a	0.13±0.01^b	0.010
14	4,7-dimethyl-undecane	1053.72	1185	17301-32-5	93.82	1.51±0.09	1.35±0.10	1.40±0.06	0.371
15	5,5-dimethyl-undecane	1162.84	1229	17312-73-1	62.35	0.26±0.02	0.23±0.03	0.21±0.02	0.218
16	3-(acetyloxymethyl)-2,2,4-trimethyl-cyclohexanol	1243.8	1530	1000162-14-5	76.03	94.30±13.90^b	178.64±7.25^a	181.89±12.51^a	0.000

17	3-nonenoic acid, methyl ester	1098.65	1191	13481-87-3	77.16	133.51±18.54[b]	232.45±3.96[a]	239.79±11.39[a]	0.000
18	butylated hydroxytoluene	1502.28	1668	128-37-0	66.72	0.16±0.03[b]	0.38±0.04[b]	0.61±0.12[a]	0.001
19	phenol	985.99	901	108-95-2	81.61	44.97±1.59	49.53±2.51	45.88±1.09	0.196
20	4-ethyl-2-methyl-phenol	1027.45	1227	2219-73-0	74.59	0.48±0.05[b]	0.69±0.07[a]	0.58±0.03[ab]	0.035
21	4-(1,1-dimethylethyl)-benzene propanal	1363.25	1508	18127-01-0	74.56	0.29±0.09[b]	0.88±0.13[a]	0.88±0.13[a]	0.002
22	nonanal	1104.68	1104	124-19-6	85.19	2.16±0.21[b]	3.05±0.15[a]	2.21±0.26[b]	0.010
23	1-(1-cyclohexen-1-yl)-1-propanone	1062.28	1126	1655-03-4	69.84	8.14±0.28[b]	9.48±0.30[a]	9.30±0.18[a]	0.002
24	2,5-hexanedione	988.47	890	110-13-4	76.05	2.52±0.08	2.80±0.12	2.58±0.08	0.103
25	4,4,5-trimethyl-2-cyclohexen-1-one	989.22	1069	17429-29-7	74.94	10.81±1.60[b]	18.24±1.08[a]	16.99±1.08[a]	0.001
26	2-heptanone	892.58	853	110-43-0	83.15	0.57±0.08[b]	1.09±0.17[a]	0.63±0.08[b]	0.009
27	2-nonanone	1091.76	1052	821-55-6	88.89	0.52±0.08[ab]	0.65±0.09[a]	0.32±0.03[b]	0.011
28	5-methyl-5-(1-methylethyl)-3-heptyne-2,6-dione	1017.17	1255	63922-44-1	69.65	0.33±0.05[b]	0.55±0.03[a]	0.51±0.03[a]	0.000
29	4-amino-2(1h)-pyridinone	1061.92	1080	38767-72-5	73.72	298.40±7.82[b]	333.46±7.96[a]	330.65±4.53[a]	0.002
30	hydrazinecarboxylic acid, ethyl ester	772.84	928	4114-31-2	79.74	30.79±0.56[a]	31.24±1.10[a]	27.70±1.06[b]	0.027
31	methyl-5-hydroxymethyl-4-imidazolecarboxylate	1192.71	1436	82032-43-7	76.93	158.64±17.91[b]	248.09±5.69[a]	244.99±9.37[a]	0.000
32	methyl dl-alpha-aminobutyrate	772.76	864	2483-62-7	63.42	2.67±0.04	2.65±0.14	2.34±0.11	0.059
33	P-aminotoluene	1155.63	1105	106-49-0	63.91	0.11±0.02[c]	0.23±0.01[b]	0.29±0.02[a]	0.000

34	4-methylmercaptoaniline	1126.71	1356	104-96-1	61.87	0.30±0.05[b]	0.55±0.04[a]	0.50±0.04[a]	0.001
35	dimethyl-sulfur diimide	1396.5	-	13849-02-0	60.0	0.08±0.02[b]	0.22±0.03[a]	0.34±0.07[a]	0.001
36	octahydro-3-methyl-1h-indole	1028.83	1188	37865-94-4	81.48	2.22±0.32[b]	3.73±0.24[a]	3.51±0.18[a]	0.001
37	1-(2-furanyl)-1-propanone	1034.44	977	3194-15-8	64.7	1.17±0.19[b]	2.22±0.13[a]	2.08±0.12[a]	0.000
38	N-(1,1-dimethylethyl)-2-benzo xazolamine	1297.23	1557	28291-84-1	75.27	0.49±0.17[b]	1.56±0.22[a]	1.79±0.29[a]	0.001
39	4,6-dimethyl-isothiazolo[5,4-b]pyridin-3-one	1478.68	1571	60750-75-6	78.78	128.47±32.09[b]	341.38±19.97[a]	379.18±40.94[a]	0.000
40	6-methyl-7-oxa-8-azabicyclo[4 .2.1]non-8-ene	1044.6	1092	1000362-10-7	75.94	3.07±0.45[b]	5.01±0.30[a]	4.64±0.28[a]	0.002
41	2-methoxy-5-methyl-thiophene	1084.39	960	31053-55-1	82.43	62.81±1.34	69.53±3.54	66.01±1.73	0.163
42	diethyl(decyloxy)-borane	1239.23	-	1000152-34-3	89.13	0.22±0.02	0.26±0.03	0.23±0.02	0.414
43	1-iodo-dodecane	1486.44	1628	4292-19-7	89.16	1.02±0.15[b]	1.72±0.13[a]	1.50±0.14[a]	0.006
44	bromo-methane	1034.71	-	74-83-9	78.45	2.36±0.39[b]	4.42±0.25[a]	4.11±0.22[a]	0.000
45	iodo-methane	1145.49	-	74-88-4	67.27	0.44±0.03[b]	0.59±0.03[a]	0.56±0.03[a]	0.002

Abbreviations: RI, retention index calculated using N-alkanes; NIST_RI: Retention index of a compound on a non-polar column; CAS, chemical abstracts service; G1, corn straw; G2, wheat shell; G3, wheat straw. The mass spectrometry fingerprints of the samples were mirrored and matched with the reference fingerprints, with high accuracy for substances with match factor > 70 and qualitative results for substances with match factor < 70.
